# Supplementary material for: Cell crowding activates pro-invasive mechanotransduction pathway in high-grade DCIS via TRPV4 inhibition and cell volume reduction
Source: eLife. 2025 Apr 21;13:RP100490. doi: 10.7554/eLife.100490 (PMC12011371; doi:10.7554/eLife.100490)
Supplement: Figure 3—source data 2. [file elife-100490-fig3-data2.zip › Figure 3B - source data 1.pdf]

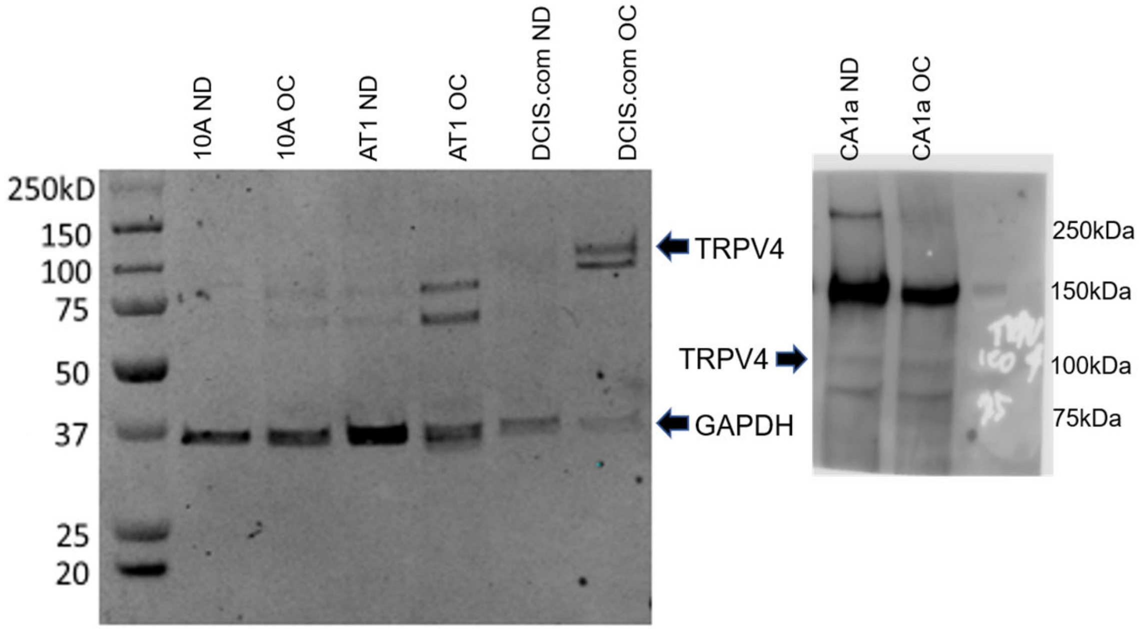


**Figure 3 – source data 1. Original immunoprecipitation and western blot images.** Left: Plasma membrane proteins pulled down after cell surface biotinylating with streptavidin beads and immunoblotted for TRPV4 and GAPDH (loading control) for ND vs OC cells of MCF10A (10A), MCF10AT1 (AT1), and MCF10DCIS.com (DCIS.com). Right: The same procedure was performed to compare PM TRPV4 between ND vs OC MCF10CA1a cells.
