# Supplementary figures and images for: Cell crowding activates pro-invasive mechanotransduction pathway in high-grade DCIS via TRPV4 inhibition and cell volume reduction
Source: eLife. 2025 Apr 21;13:RP100490. doi: 10.7554/eLife.100490 (PMC12011371; doi:10.7554/eLife.100490)

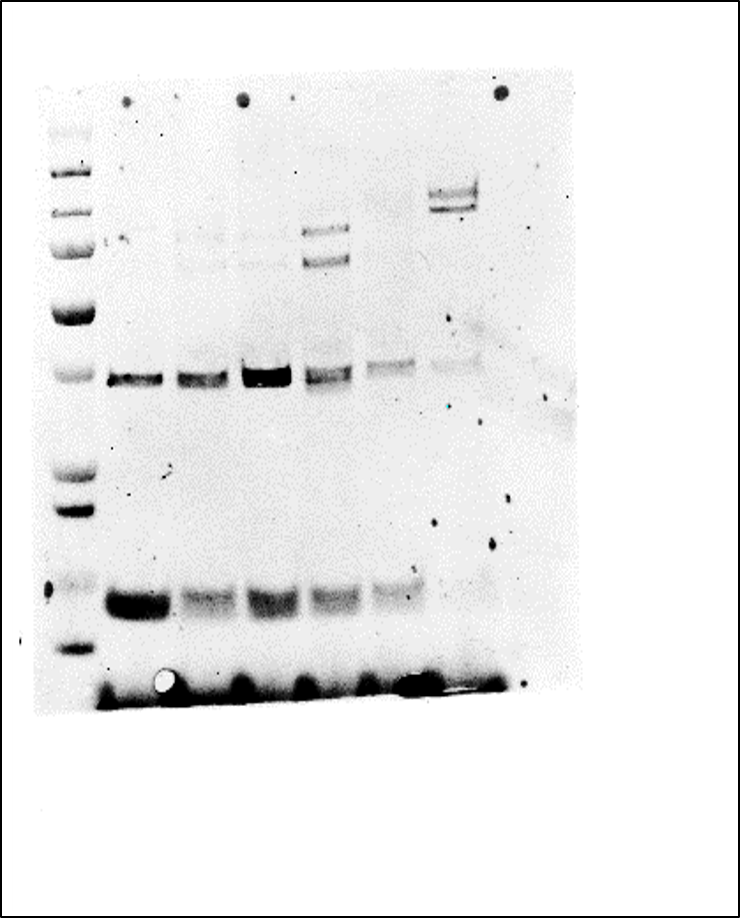

Supplement: Figure 3—source data 3. [file elife-100490-fig3-data3.zip › Figure 3B - source data 2.tif]

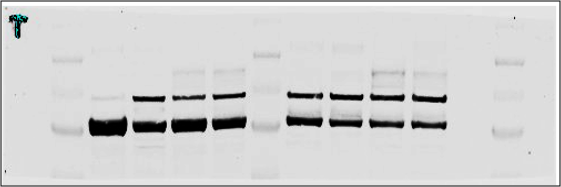

Supplement: Figure 3—source data 5. [file elife-100490-fig3-data5.zip › Figure 3C - source data 2.tif]

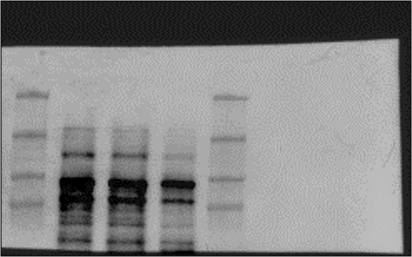

Supplement: Figure 3—figure supplement 1—source data 2. [file elife-100490-fig3-figsupp1-data2.zip › Figure 3 - figure supplement 1B - source data 2.tif]
