# Supplementary material for: Cell crowding activates pro-invasive mechanotransduction pathway in high-grade DCIS via TRPV4 inhibition and cell volume reduction
Source: eLife. 2025 Apr 21;13:RP100490. doi: 10.7554/eLife.100490 (PMC12011371; doi:10.7554/eLife.100490)
Supplement: Figure 3—source data 4. [file elife-100490-fig3-data4.zip › Figure 3C - source data 1.pdf]

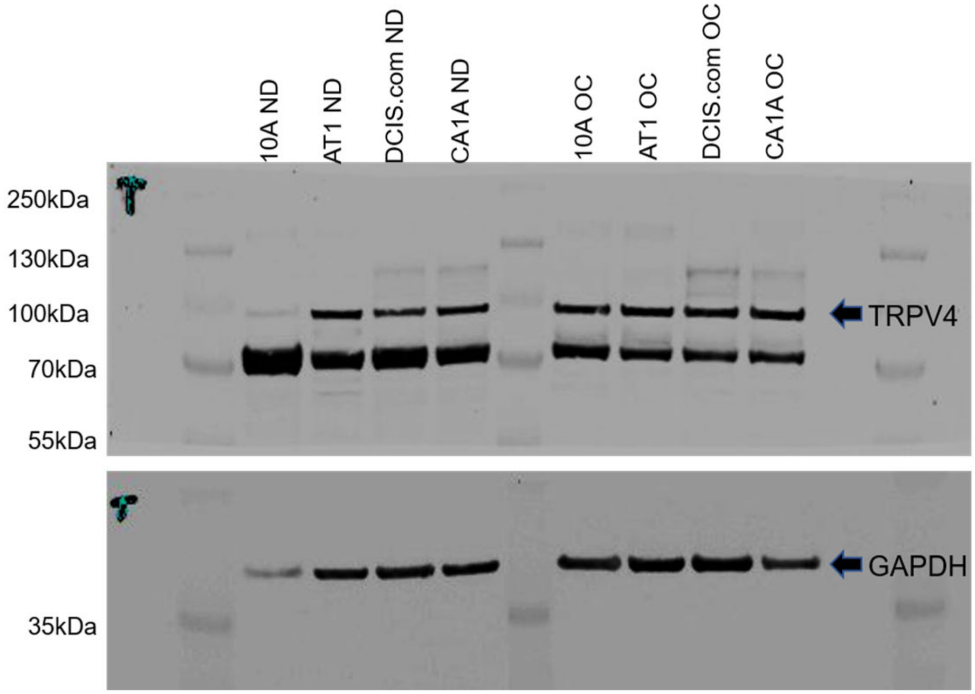

Figure 3C – source data 1. Original western blot images. Overall TRPV4 protein levels from whole-cell lysates from four 10A cell derivatives. GAPDH was a loading control.
