# Supplementary material for: Cell crowding activates pro-invasive mechanotransduction pathway in high-grade DCIS via TRPV4 inhibition and cell volume reduction
Source: eLife. 2025 Apr 21;13:RP100490. doi: 10.7554/eLife.100490 (PMC12011371; doi:10.7554/eLife.100490)
Supplement: Figure 3—source data 7. — Ion channels among these are highlighted in bold. [file elife-100490-fig3-data7.pdf]

**Gene names MCF10DCIS.com cells**	**Protein Name (MCF10DCIS.com)**	**OC/ND ratio (MCF10DCIS.com)**
**TRPV4**	Transient Receptor Potential Vanilloid 4	153.01
IVL	Involucrin	55.35
**SCN11A;SCN5A;SCN10A;SCN9A**	Sodium Voltage-Gated Channel Alpha Subunit 11; Sodium Voltage-Gated Channel Alpha Subunit 5; Sodium Voltage-Gated Channel Alpha Subunit 10; Sodium Voltage-Gated Channel Alpha Subunit 9	41.82
HNRNPM	Heterogeneous Nuclear Ribonucleoprotein M	26.91
COPG2	Coatomer Protein Complex Subunit Gamma 2	18.33
S100P	S100 calcium-binding protein P	18.01
MRPL16	Mitochondrial Ribosomal Protein L16	14.43
IDH2	Isocitrate Dehydrogenase [NADP] Mitochondrial	8.68
EML4	Echinoderm Microtubule-Associated Protein-Like 4	7.94
GSN	Gelsolin	7.89
CKAP4	Cytoskeleton-Associated Protein 4	7.82
PRSS8	Serine Protease 8	6.93
MFI2	Melanotransferrin	6.85
CEACAM1	Carcinoembryonic Antigen-Related Cell Adhesion Molecule 1	6.84
ADGRL3;LPHN3	Adhesion G protein-coupled receptor L3; Latrophilin-3	6.82
HNRNPC	Heterogeneous Nuclear Ribonucleoprotein C	6.78
RPL7L1	Ribosomal Protein L7-Like 1	6.54

NDUFV1	NADH:Ubiquinone Oxidoreductase Core Subunit V1	6.19
HIST1H2A;HIST1H2AH;HIST1H2AF;HIST1H2AJ;HIST1H2AG;HIST1H2AA3;HIST2H2AA3;H2AFX	Histone H2A family	6.07
GTF2I	General Transcription Factor II-I	5.88
ERP44	Endoplasmic Reticulum Protein 44	5.81
**KCNN4**	Potassium Intermediate Conductance Calcium-Activated Channel 4	5.61
NDUFB9	NADH:Ubiquinone Oxidoreductase Subunit B9	5.59
RPL32	Ribosomal Protein L32	5.47

Figure 3 – source data 7. Gene and protein names that showed more than a 5-fold increase in plasma membrane association under OC conditions relative to ND conditions in MCF10DCIS.com cells were identified by mass spectrometry. Ion channels among these are highlighted in bold.
