# Supplementary material for: Cell crowding activates pro-invasive mechanotransduction pathway in high-grade DCIS via TRPV4 inhibition and cell volume reduction
Source: eLife. 2025 Apr 21;13:RP100490. doi: 10.7554/eLife.100490 (PMC12011371; doi:10.7554/eLife.100490)
Supplement: Figure 3—source data 8. — One ion transporter showing plasma membrane relocation under OC conditions in MCF10CA1a cells is highlighted in bold. [file elife-100490-fig3-data8.pdf]

Gene MCF10A cells	Protein Name (MCF10A)	OC/ND ratio (MCF10A)	Gene MCF10AT1 cells	Protein Name (MCF10AT1)	OC/ND ratio (MCF10AT1)	Gene MCF10CA1a cells	Protein Name (MCF10CA1a)	OC/ND ratio (MCF10CA1a)
SCEL	Scellicin	5318.64	HTRA2	High temperature requirement A2	7.36	ACAA2	Acetyl-CoA acyltransferase 2	82.61
ALDH7A1	Aldehyde dehydrogenase 7 family member A1	3111.33	PMPCB	Peptidase (mitochondrial processing) beta	5.86	**ATP2B4**	Plasma membrane calcium-transporting ATPase 4	37.51
ECH1	Enoyl CoA hydratase 1	2711.84	CLPP	ATP-dependent Clp protease proteolytic subunit	5.58	LETM1	Leucine zipper-EF-hand containing transmembrane protein 1	33.35
TRAP1	TNF Receptor Associated Protein 1	1730.73				PCCB	Propionyl-CoA carboxylase beta chain	31.27
ACAA2	Acetyl-CoA acyltransferase 2	1220.09				CEACAM5	Carcinoembryonic antigen-related cell adhesion molecule 5	24.58
AK3	Adenylate kinase 3	981.946				PTPRF	Protein tyrosine phosphatase, receptor type F	21.94
UQCRC1	Ubiquinol-cytochrome c reductase core protein I	854.604				MST1R	Macrophage stimulating 1 receptor	21.13
DCXR	Dicarbonyl/L-xylulose reductase	819.989				CNNM3	Cyclin and CBS domain divalent metal cation transport mediator 3	16.02
SSBP1	Single-stranded DNA-binding protein 1	808.072				SLC27A4	Fatty acid transporter 4	14.53

NDUFB6	NADH dehydrogenase [ubiquinone] 1 beta subcomplex 6	757.227				CD70	CD70 molecule	14.45
S100A14	S100 calcium-binding protein A14	725.033				ST14	Suppression of tumorigenicity 14 (serine peptidase)	13.58
S100A9	S100 calcium-binding protein A9	680.512				ECHS1	Enoyl-CoA hydratase, short chain 1	13.28
S100A8	S100 calcium-binding protein A8	621.973				PVRL4	Poliovirus receptor-related protein 4	13.12
CYB5R1	Cytochrome b5 reductase 1	583.052				PTPRK	Protein tyrosine phosphatase, receptor type K	12.76
EPHX1	Epoxide hydrolase 1	581.606				SPINT1	Serine peptidase inhibitor, Kunitz type 1	12.69
NDUFV1	NADH dehydrogenase [ubiquinone] flavoprotein 1	558.186				ROBO1	Roundabout Guidance Receptor 1	12.38
COX5A	Cytochrome c oxidase subunit 5A	558.156				NIPSNAP1	Nipsnap homolog 1	9.31
SOD2	Superoxide dismutase 2	458.521				ITGA5	Integrin subunit alpha 5	9.23
GRHPR	Glyoxylate reductase/hydroxypyruvate reductase	440.284				ITGAV	Integrin subunit alpha V	8.62
SDHA	Succinate dehydrogenase complex, subunit A	393.879				GOT2	Glutamate oxaloacetate transaminase 2	8.54
CS	Citrate synthase	381.161				ALCAM	Activated leukocyte cell adhesion molecule	7.74
HSD17B10	Hydroxysteroid (17-beta) dehydrogenase 10	369.796				TUBB2B,TUBB2A	Tubulin beta 2B class IIb; Tubulin beta 2A class IIa	7.7

ETFB	Electron transfer flavoprotein subunit beta	332.828				PSAP	Prosaposin	7.51
NDUFA10	NADH dehydrogenase [ubiquinone] 1 alpha subcomplex 10	312.164				SLC27A1	Fatty acid transporter 1	7.22

Figure 3 – source data 8. Top 25 genes and corresponding proteins that exhibited more than a 100-fold increase in plasma membrane association under OC conditions compared to ND conditions in MCF10A cells (left), and more than a 5-fold increase in MCF10AT1 (middle) and MCF10CA1a (right) cells, as identified by mass spectrometry. One ion transporter showing plasma membrane relocation under OC conditions in MCF10CA1a cells is highlighted in bold.
