# Supplementary material for: Cell crowding activates pro-invasive mechanotransduction pathway in high-grade DCIS via TRPV4 inhibition and cell volume reduction
Source: eLife. 2025 Apr 21;13:RP100490. doi: 10.7554/eLife.100490 (PMC12011371; doi:10.7554/eLife.100490)
Supplement: Figure 3—figure supplement 1—source data 1. [file elife-100490-fig3-figsupp1-data1.zip › Figure 3 - figure supplement 1B - source data 1.pdf]

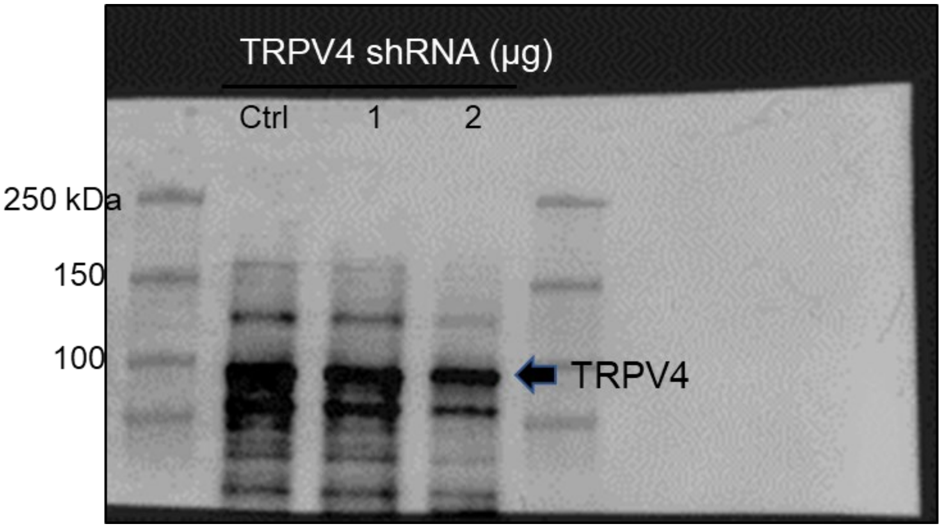

Figure 3 – figure supplement 1B – source data 1. Immunoblot results confirmed this dose-responsive depletion of TRPV4, with 33% reduction observed at 1 µg shRNA and 51% at 2 µg.
