# Supplementary material for: Cell crowding activates pro-invasive mechanotransduction pathway in high-grade DCIS via TRPV4 inhibition and cell volume reduction
Source: eLife. 2025 Apr 21;13:RP100490. doi: 10.7554/eLife.100490 (PMC12011371; doi:10.7554/eLife.100490)
Supplement: Figure 3—figure supplement 6—source data 1. [file elife-100490-fig3-figsupp6-data1.pdf]

ROI Images

0. Normal
1. Benign
2. ADH
3. Low-DCIS
4. IMG-DCIS
5. HG-DCIS
6. IMG-IDC
7. HG-IDC

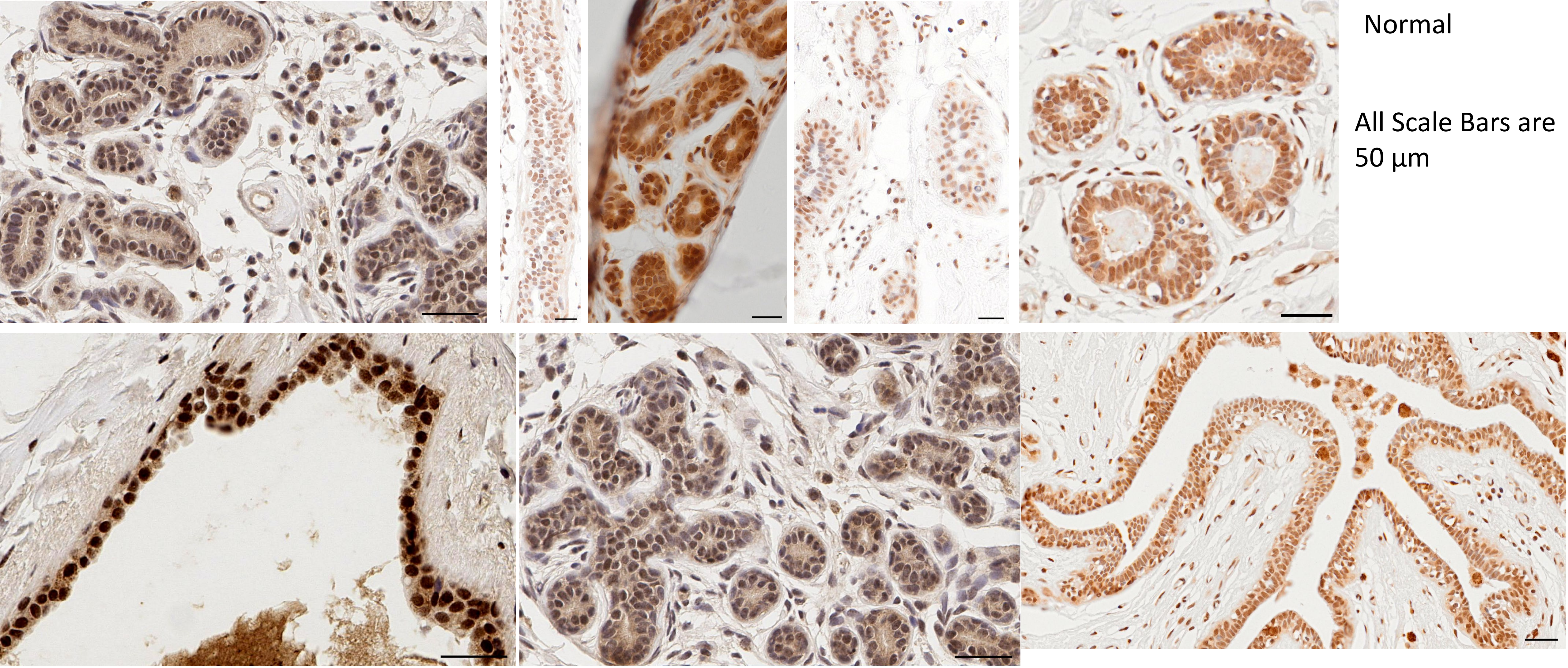

Normal

All Scale Bars are 50 μm

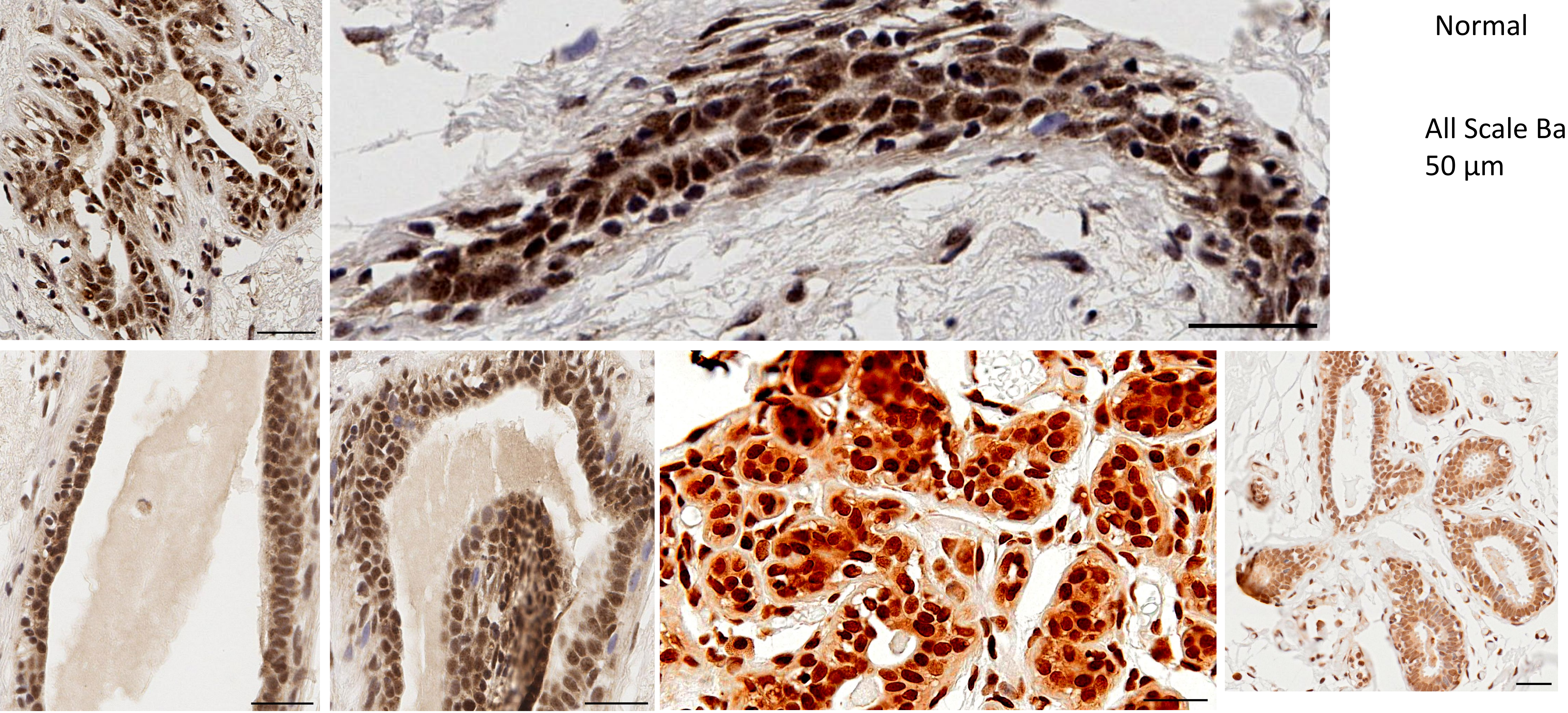

Normal

All Scale Bars are 50 µm

Benign

All Scale Bars are 50 µm

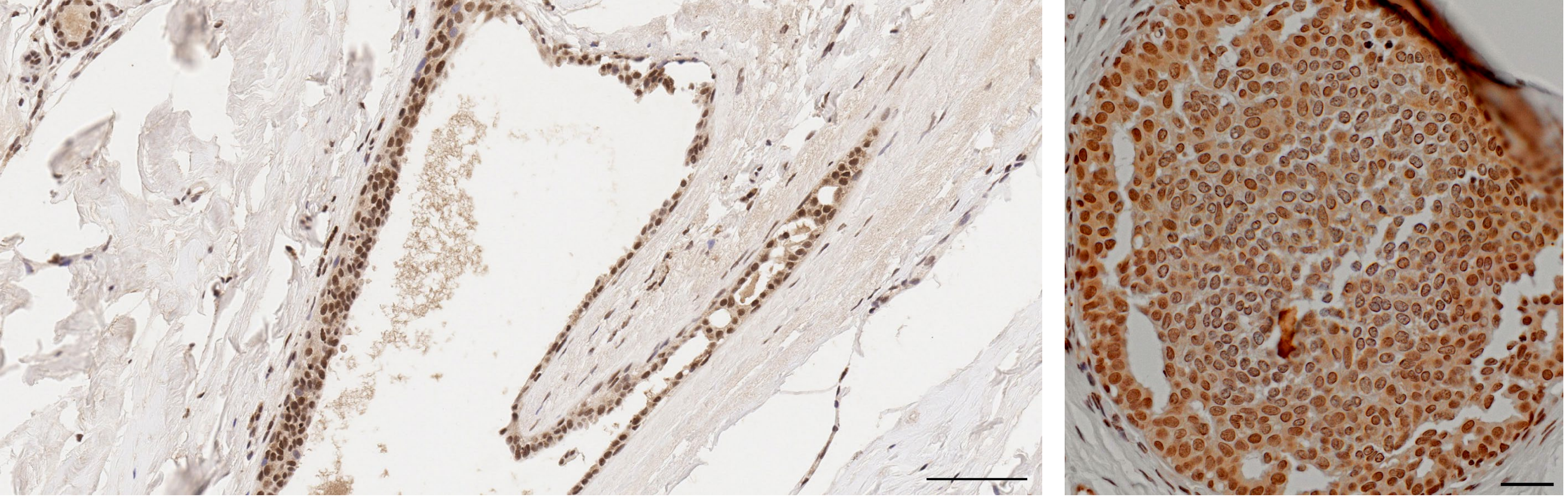

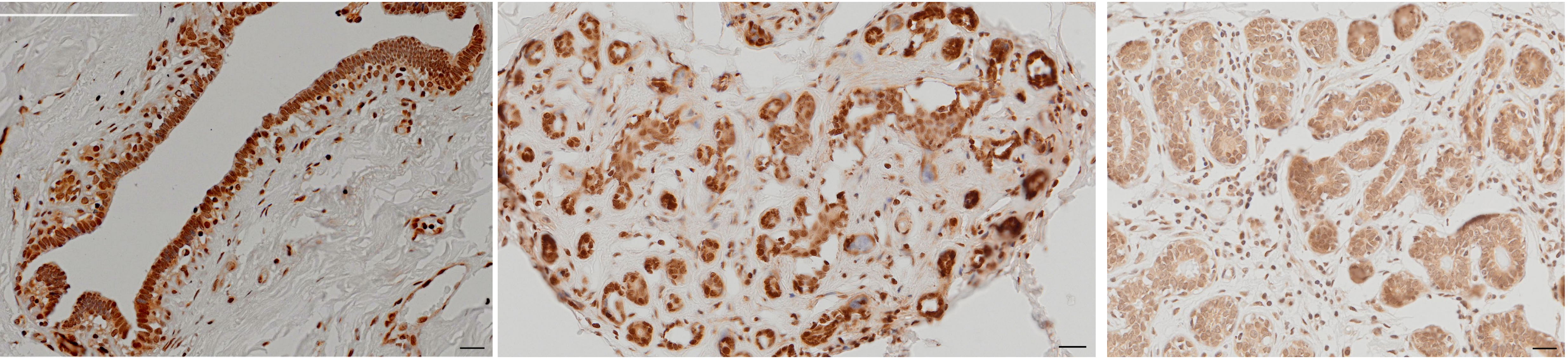

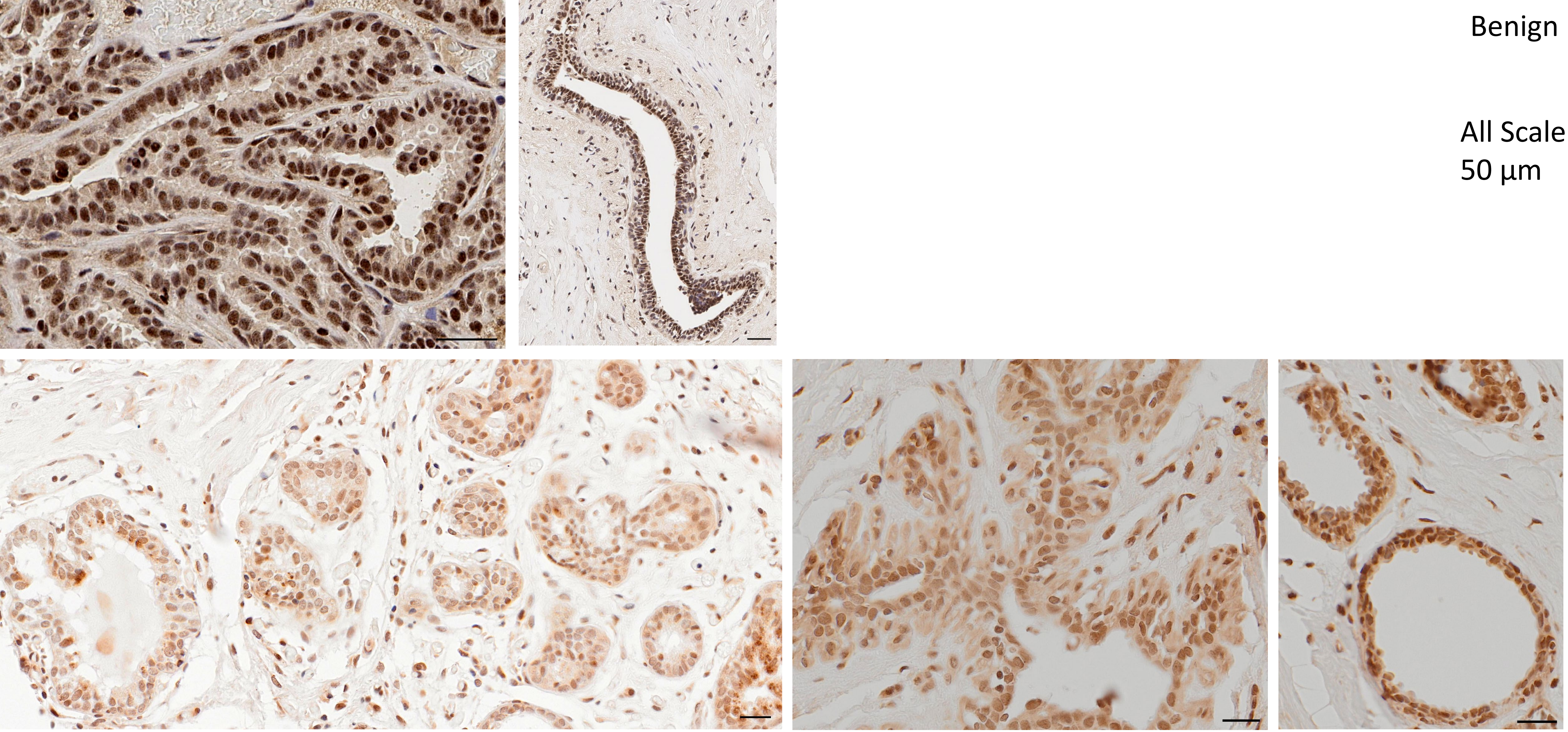

Benign

All Scale Bars are 50 µm

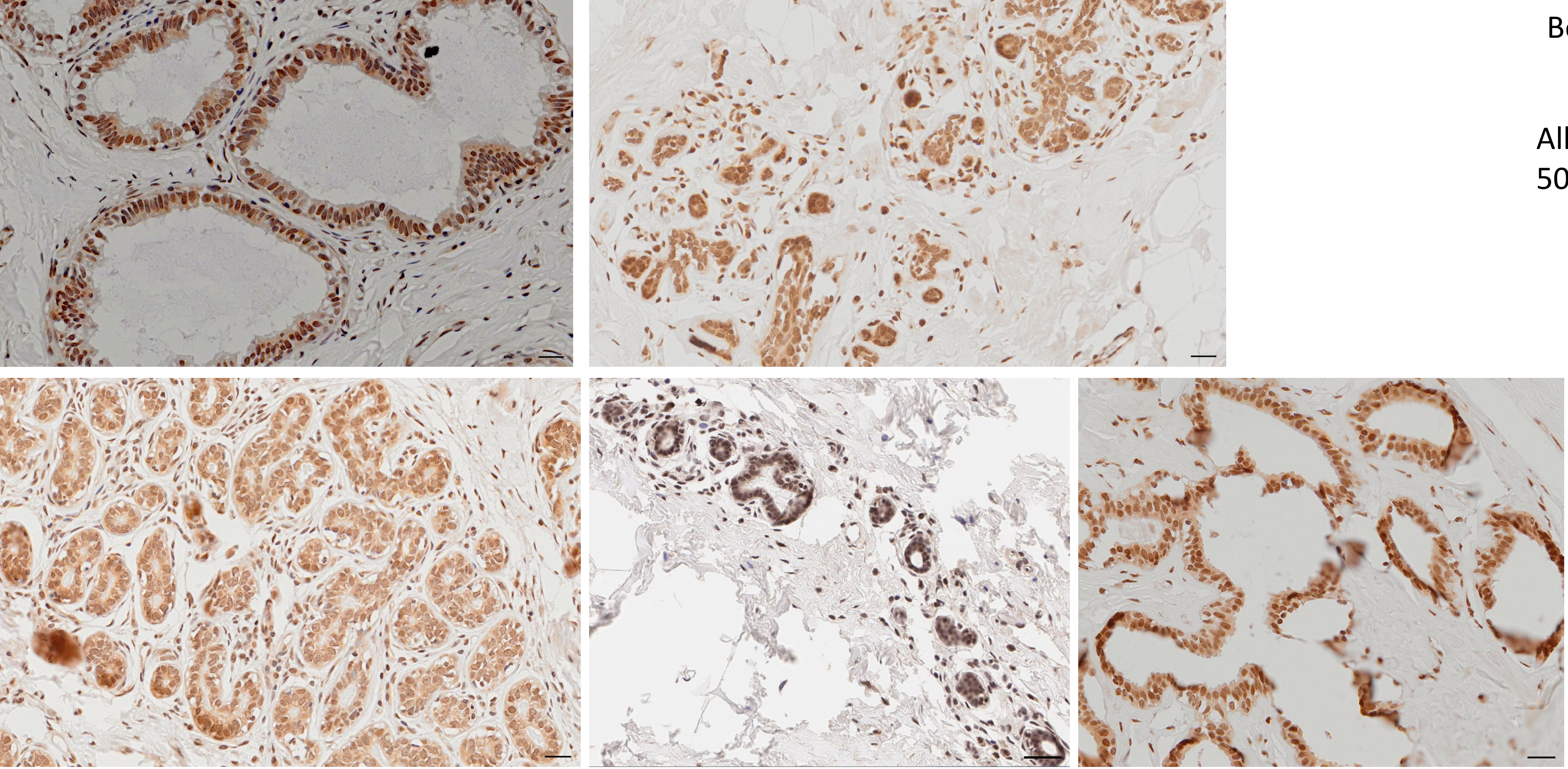

Benign

All Scale Bars are 50 µm

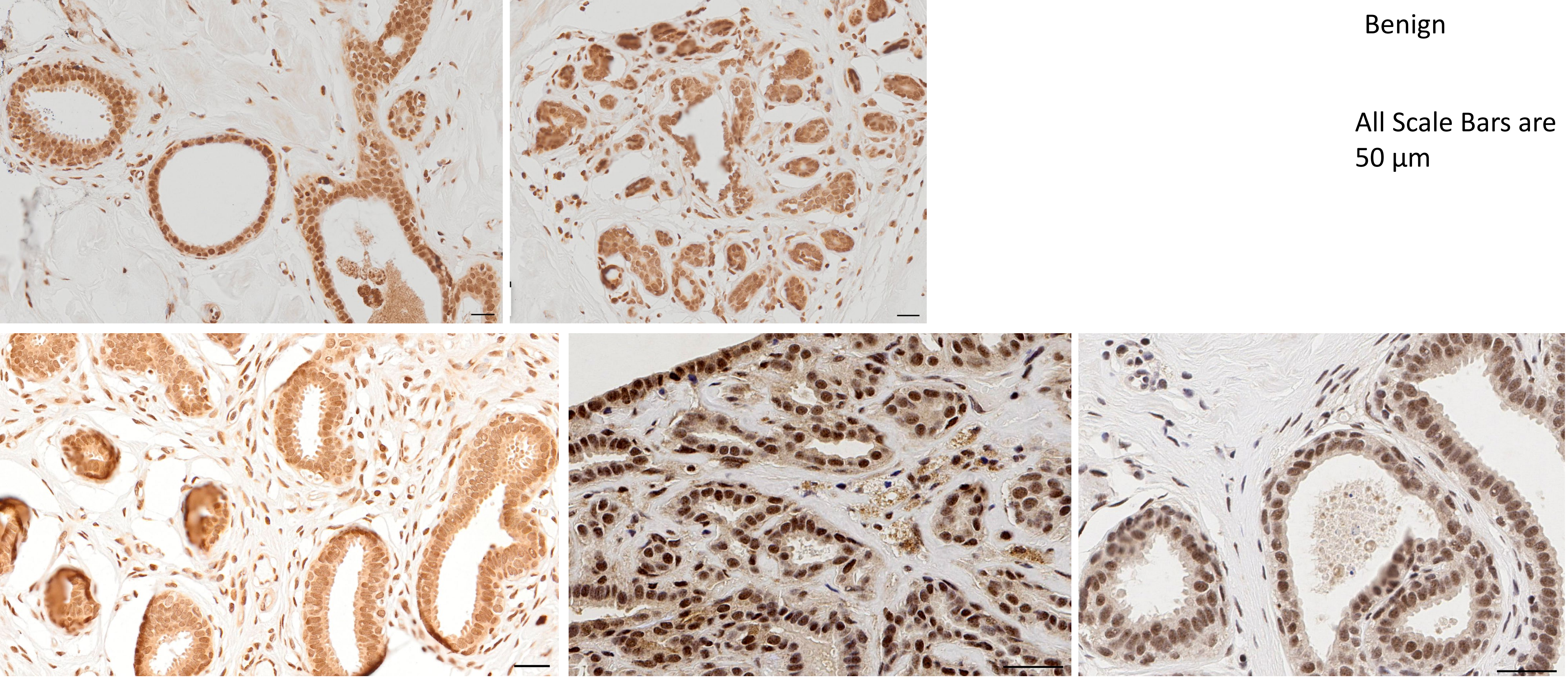

Benign

All Scale Bars are 50 µm

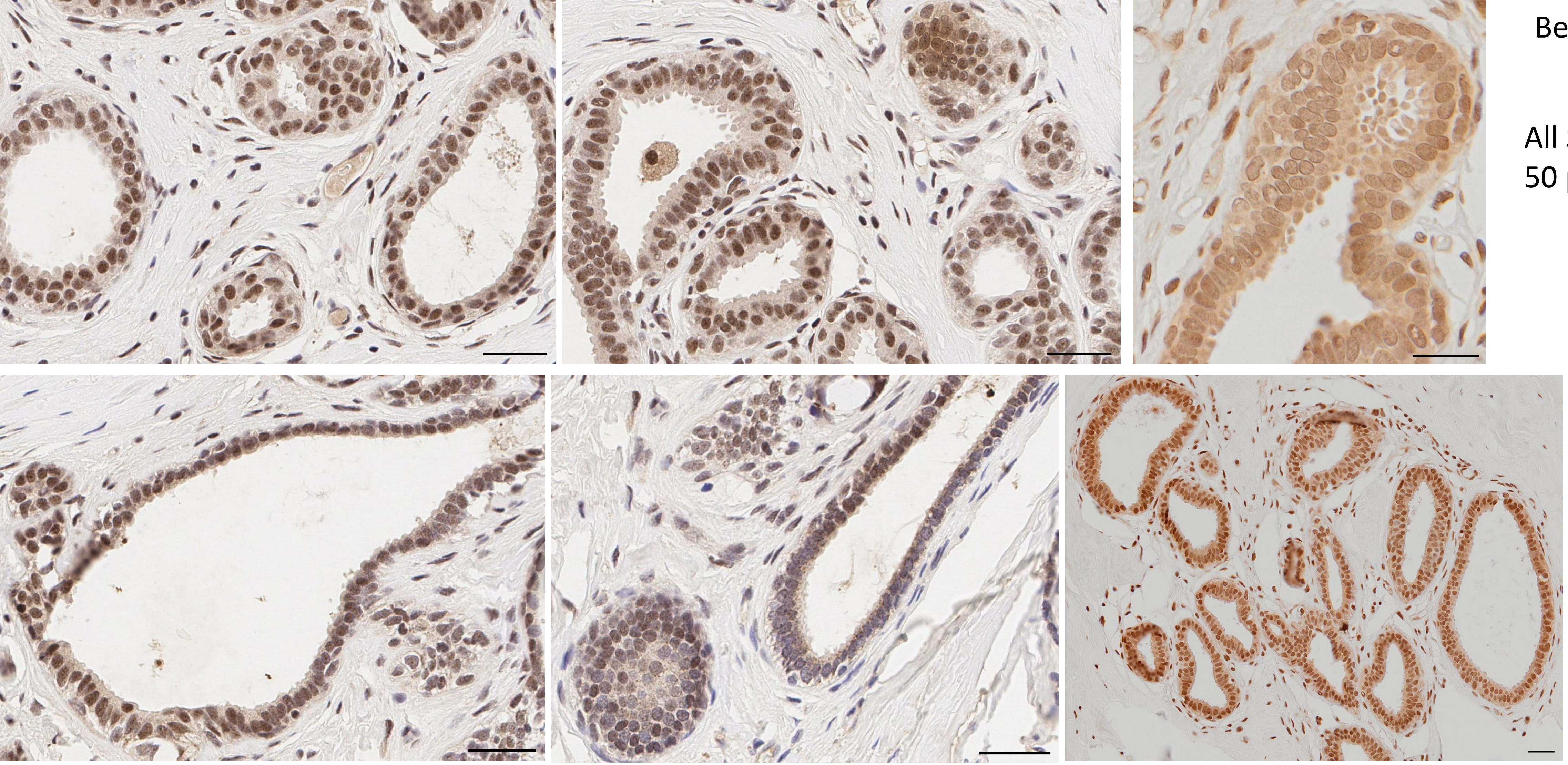

Benign

All Scale Bars are 50 µm

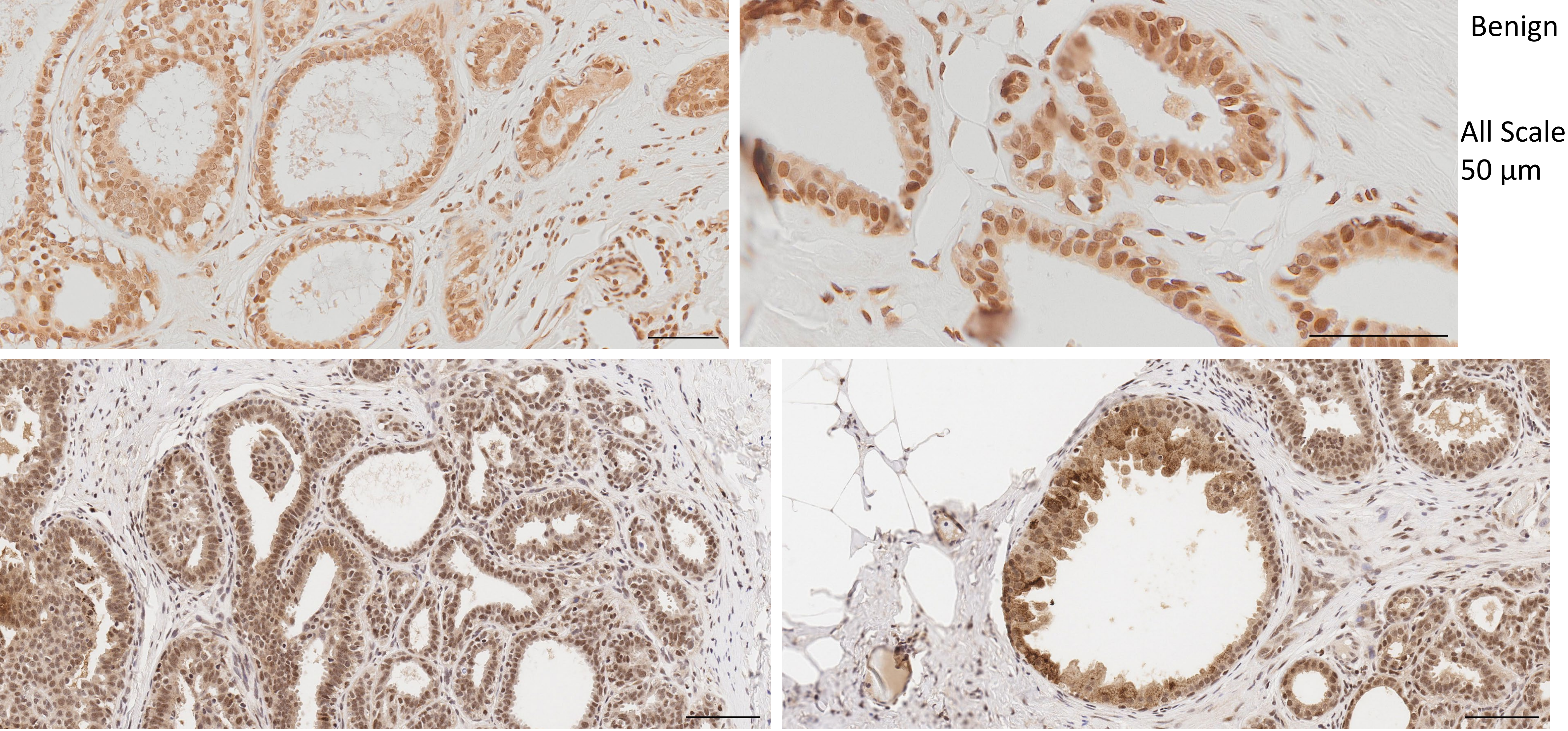

Benign

All Scale Bars are 50 µm

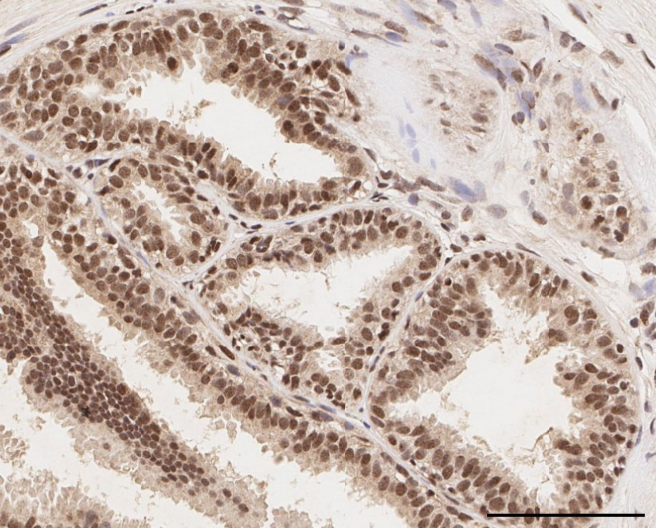

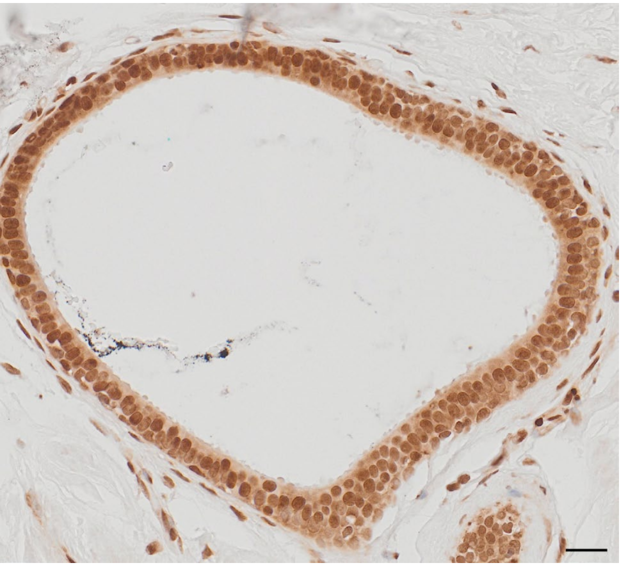

Benign

All Scale Bars are
50 µm

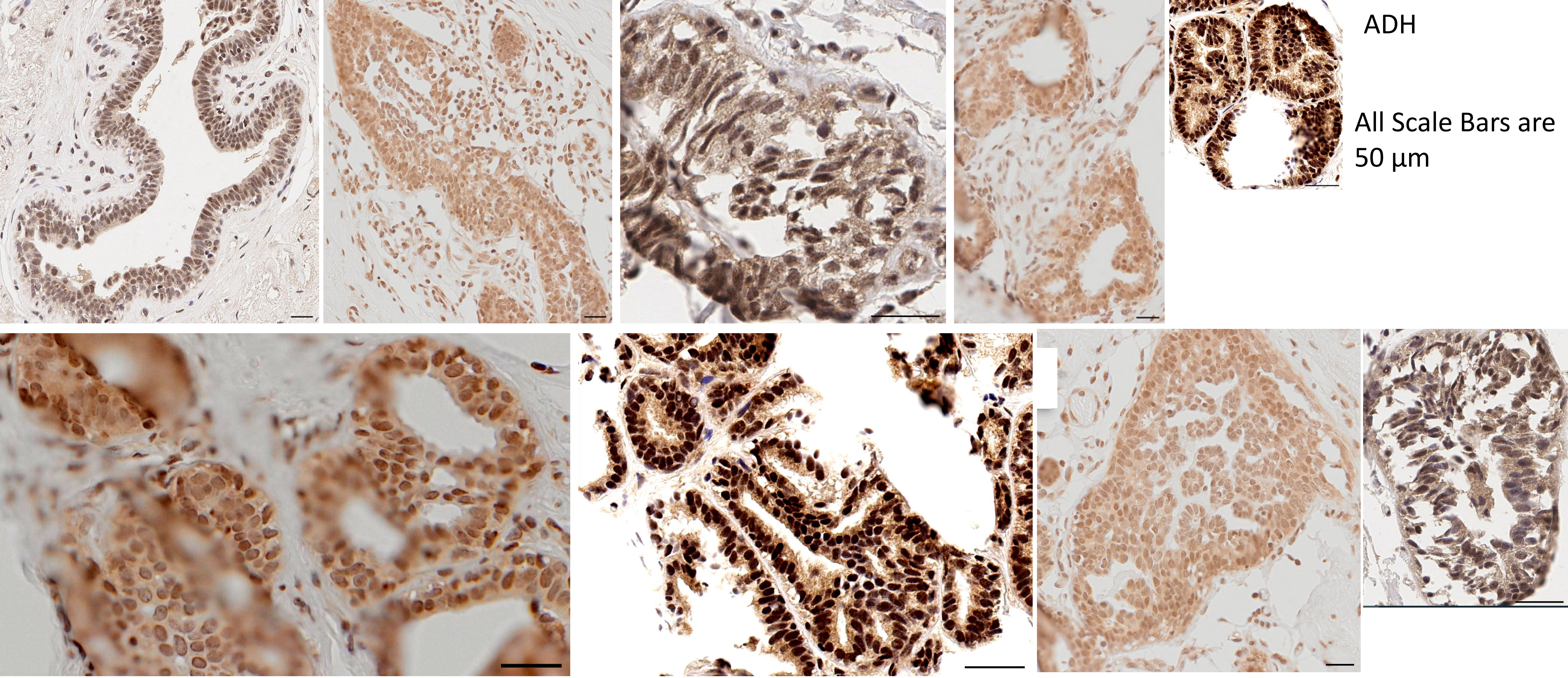
ADH
All Scale Bars are 50 µm

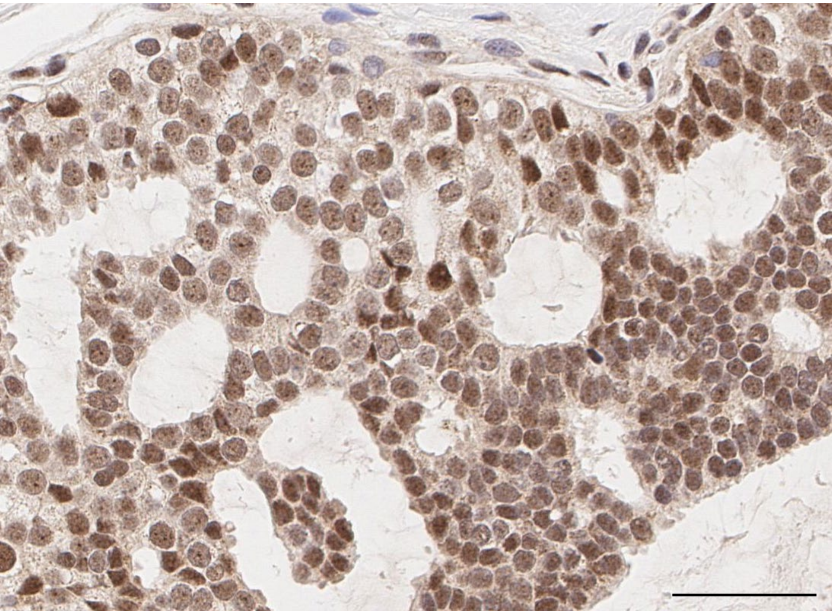

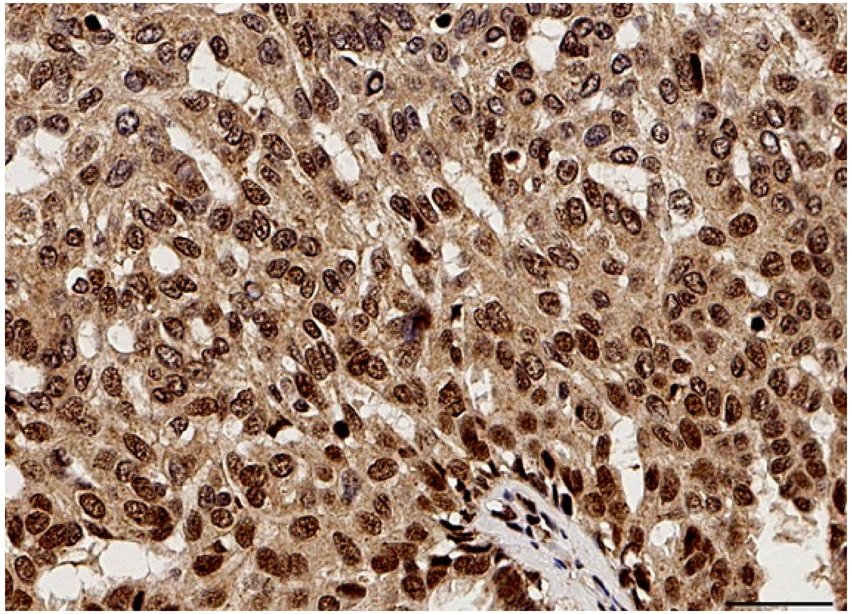

Low Grade DCIS

All Scale Bars are 50 µm

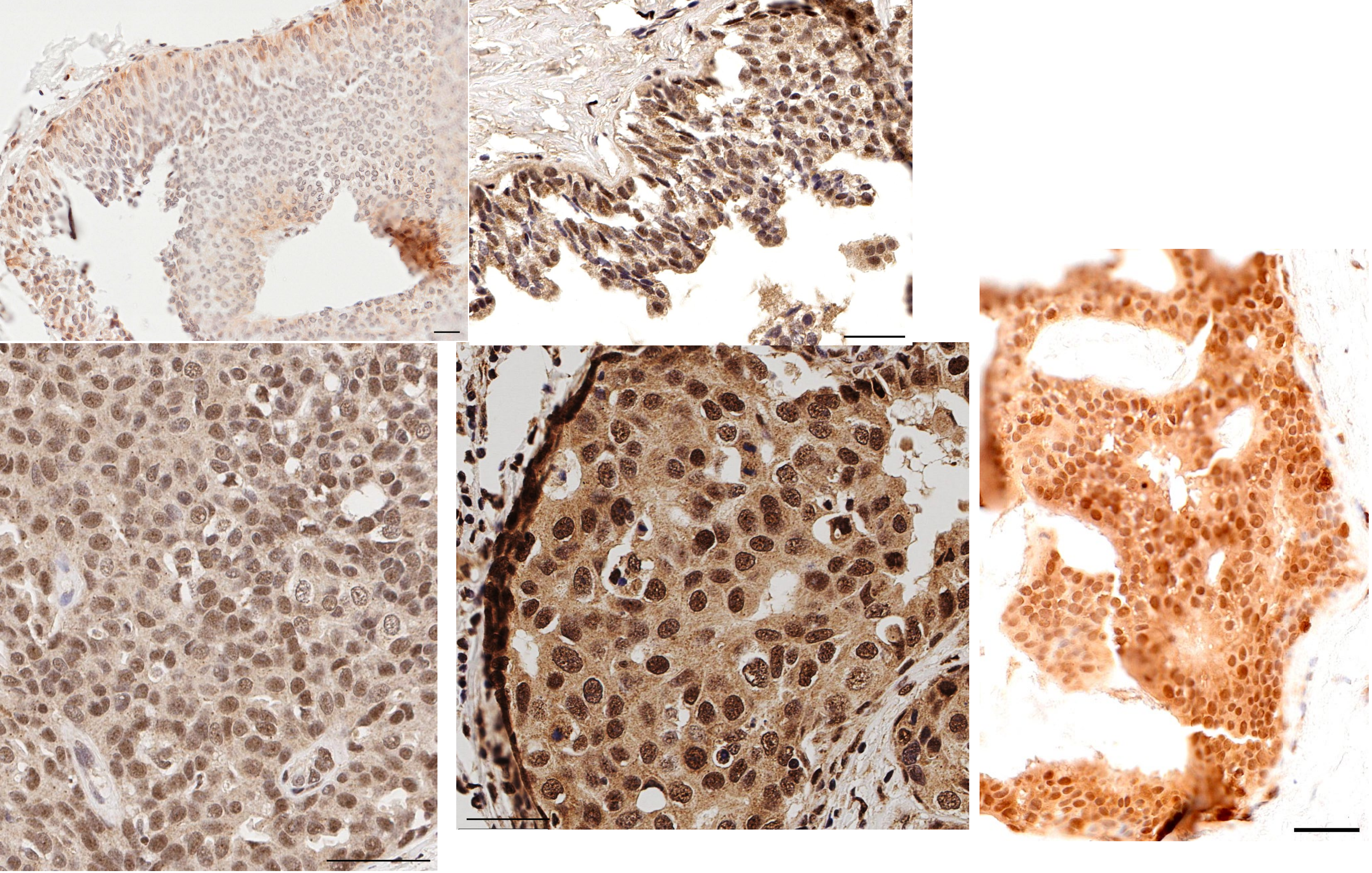

Intermediate Grade DCIS

All Scale Bars are 50 µm

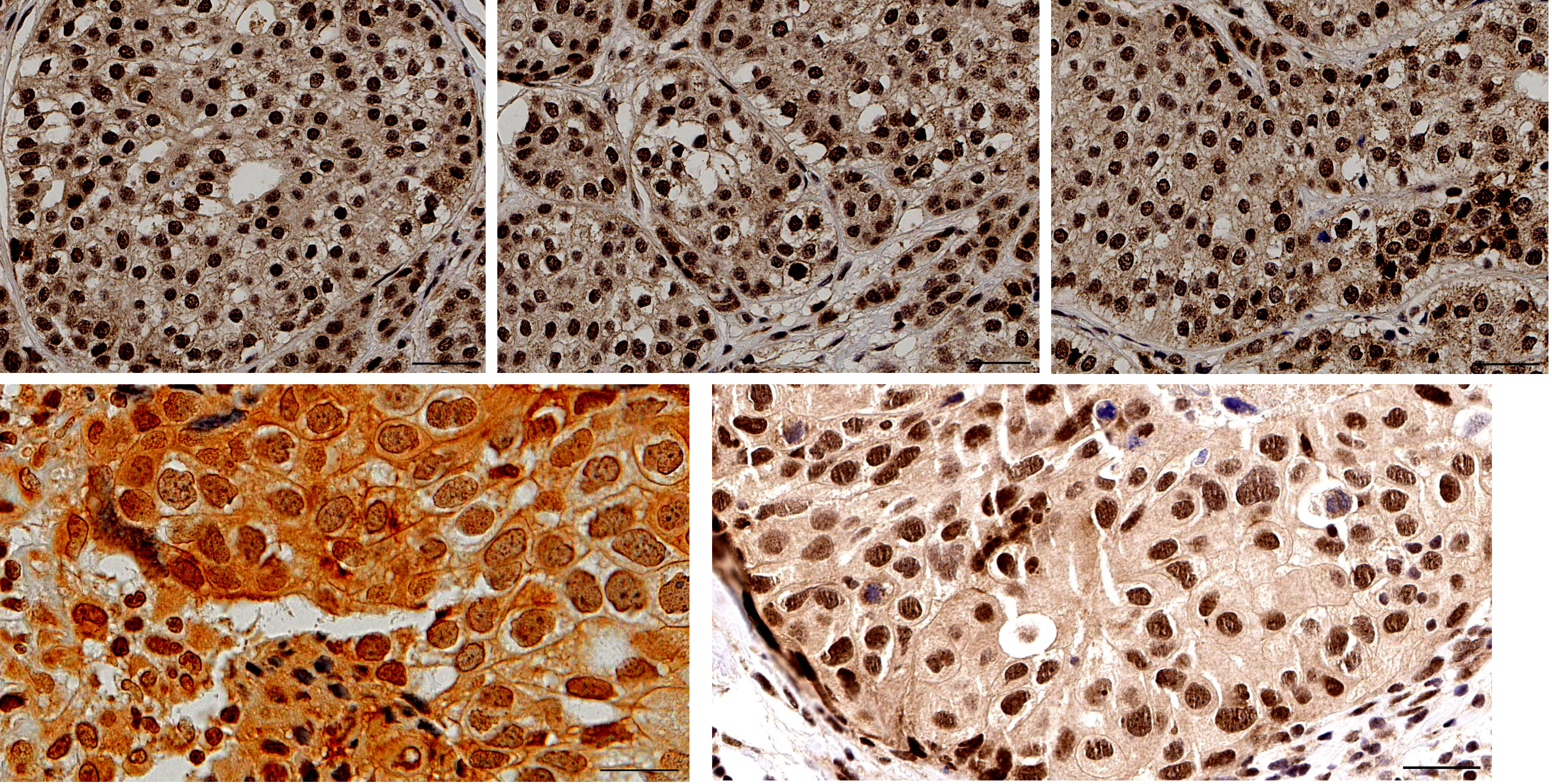

High Grade DCIS

All Scale Bars are 50 µm

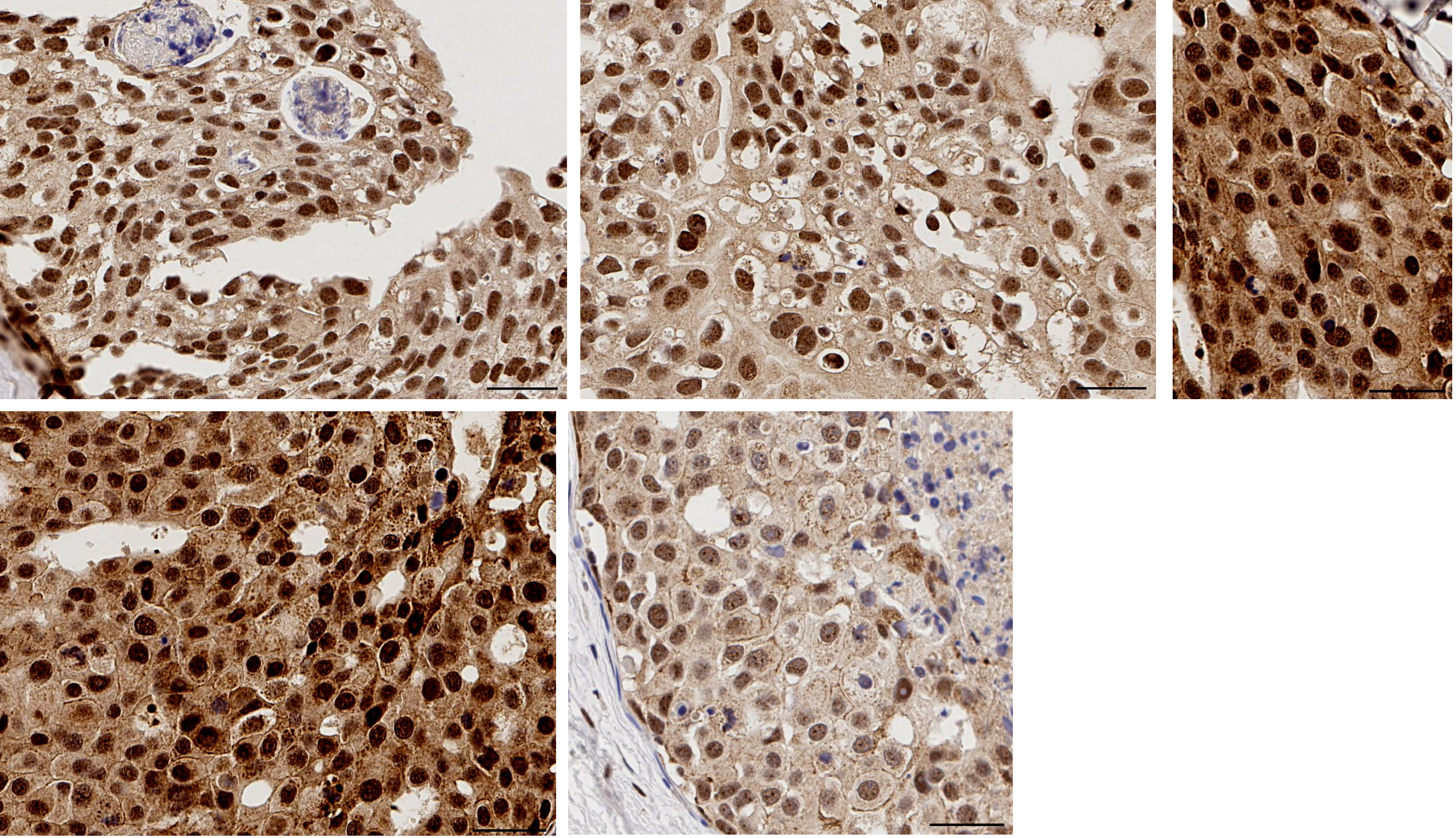

High Grade DCIS

All Scale Bars are 50 μm

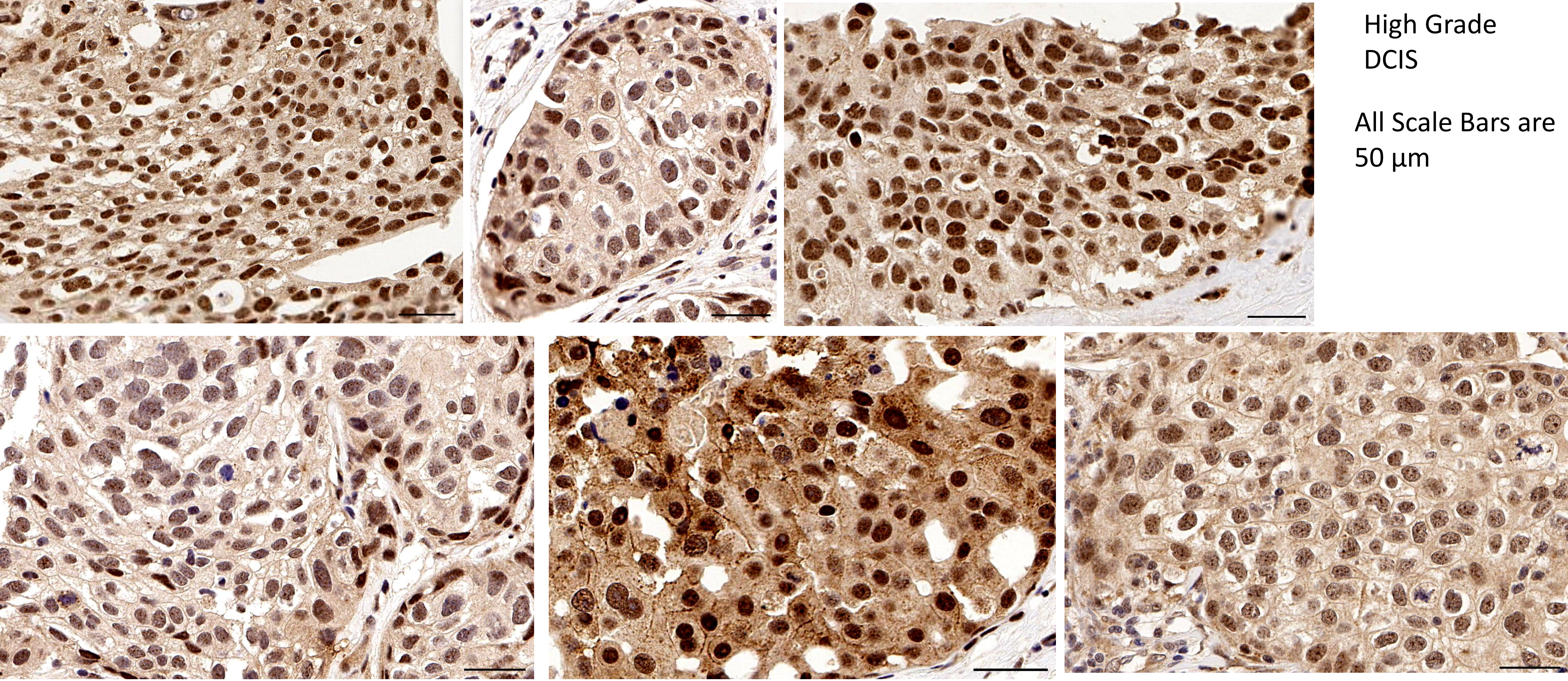

High Grade DCIS

All Scale Bars are 50 µm

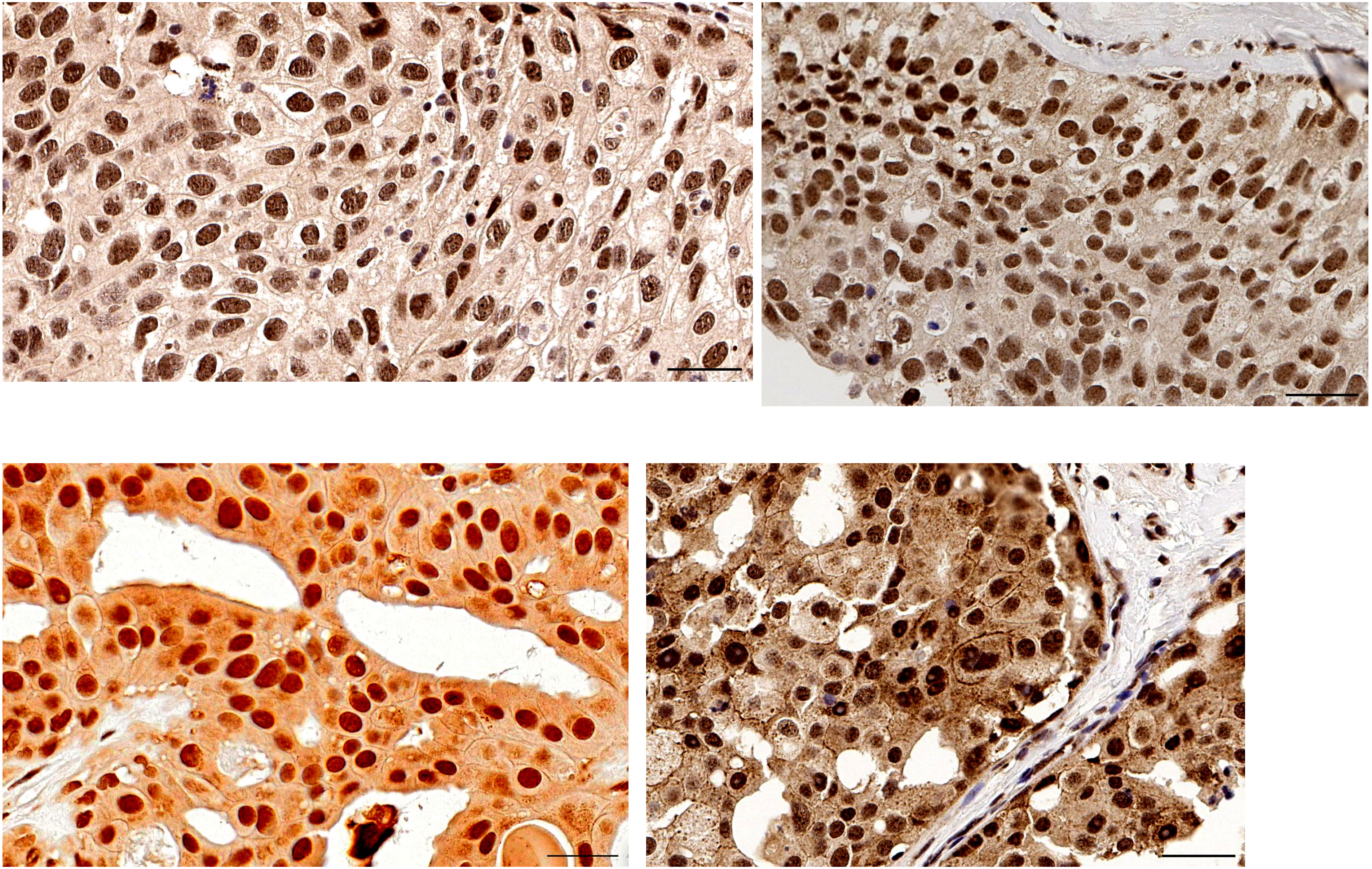

High Grade
DCIS

All Scale Bars are
50 µm

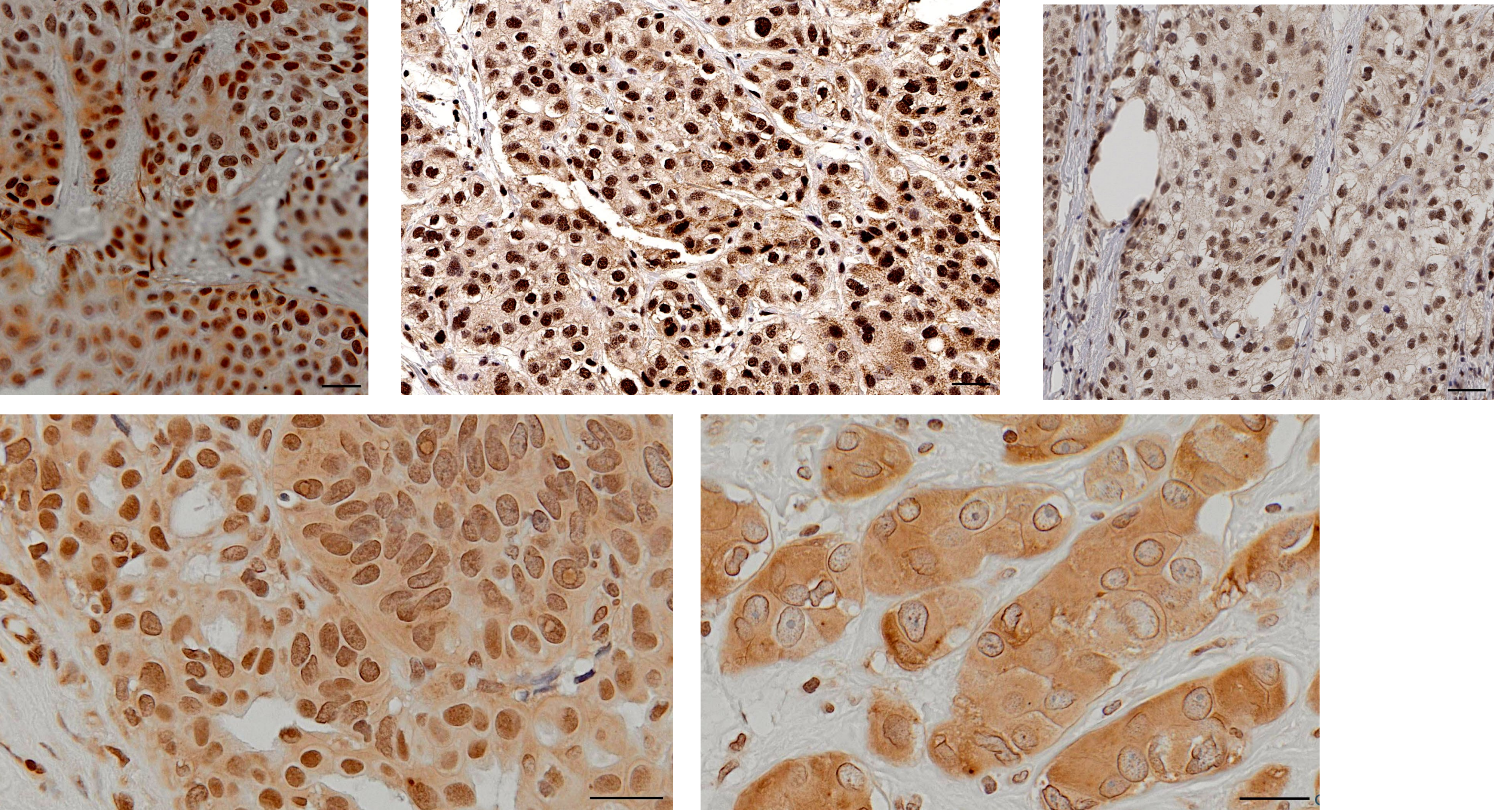

IDC

All Scale Bars are 50 µm

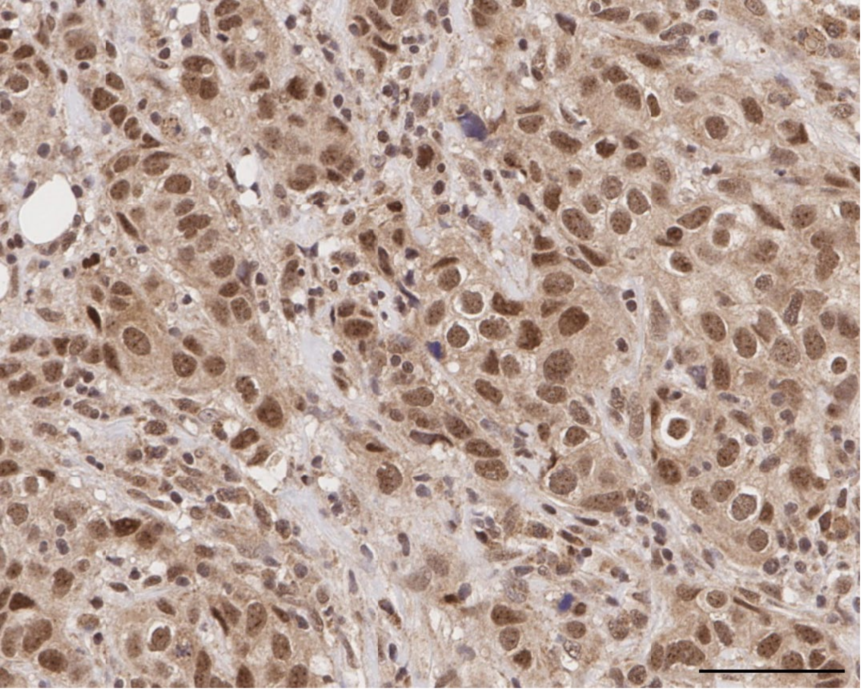

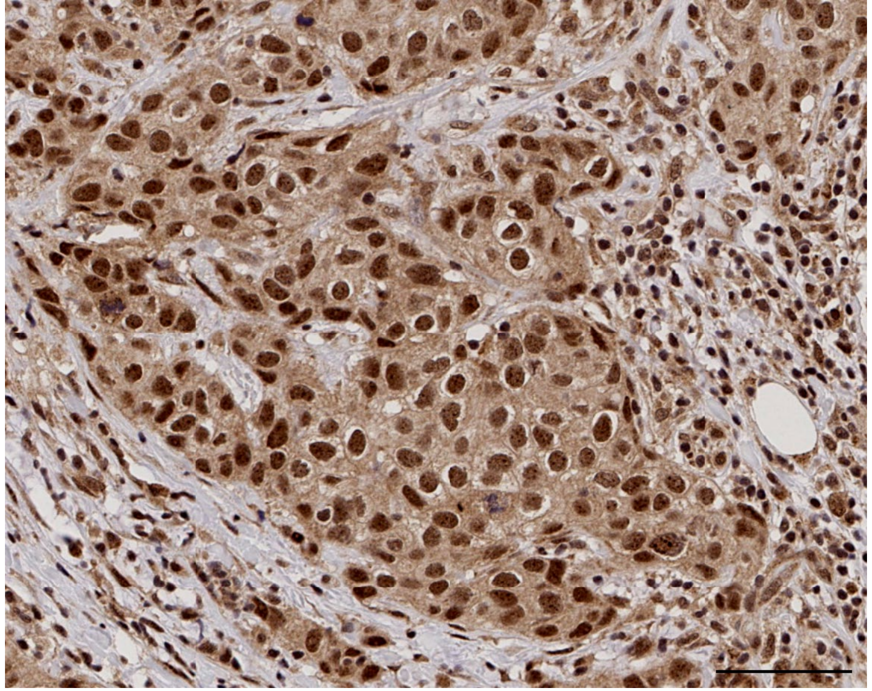

IDC

All Scale Bars are 50 µm

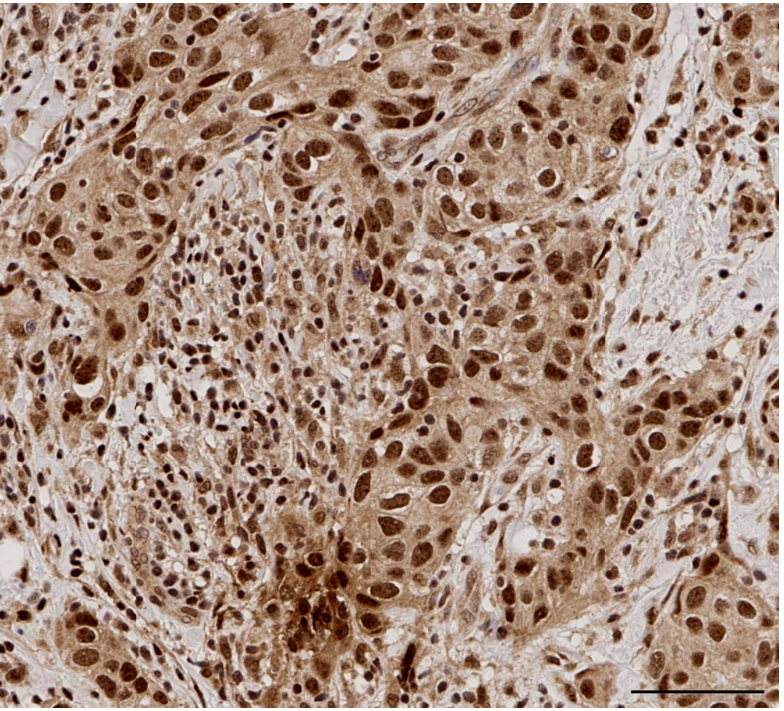

IDC

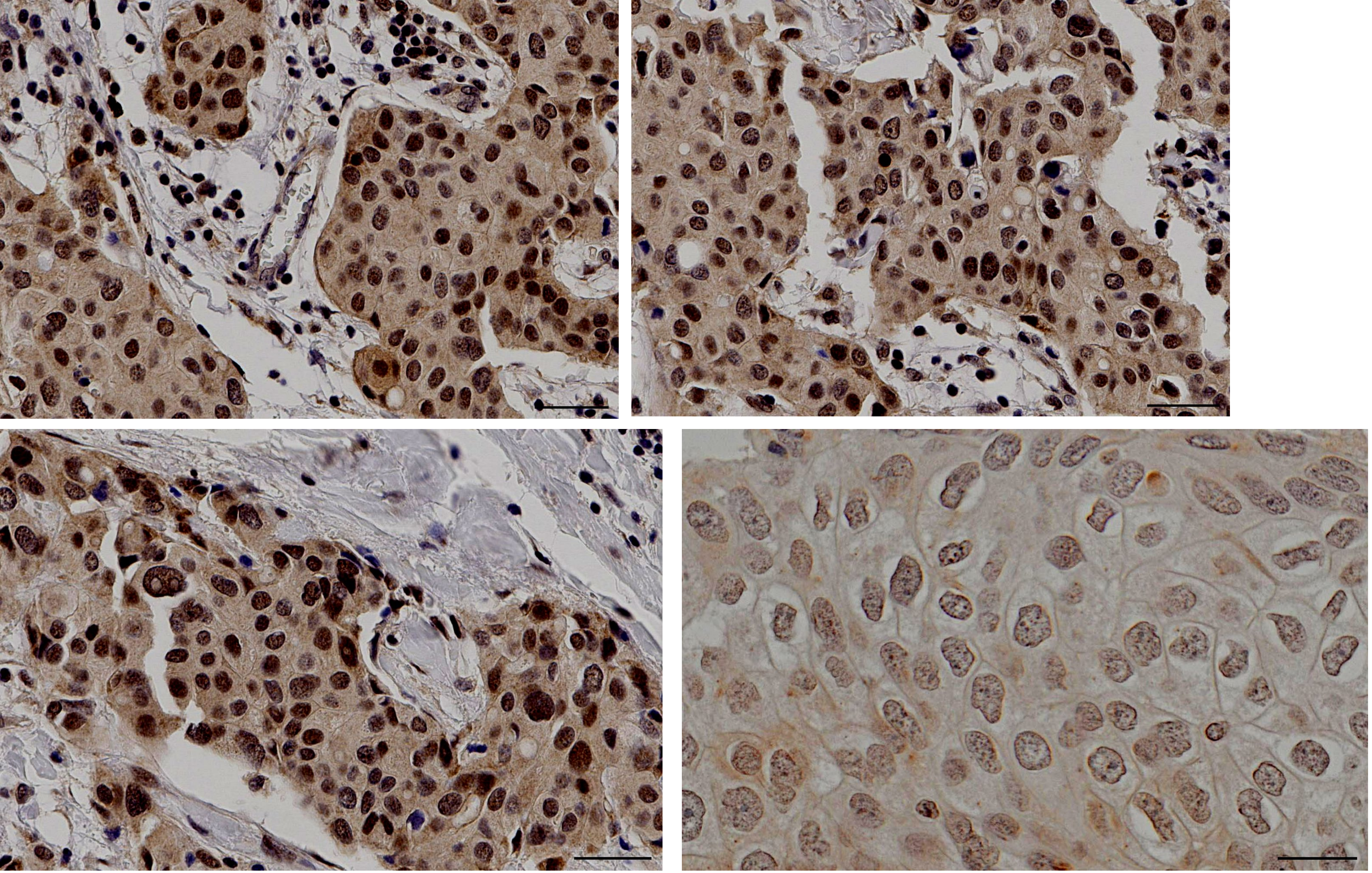

All Scale Bars are 50 µm

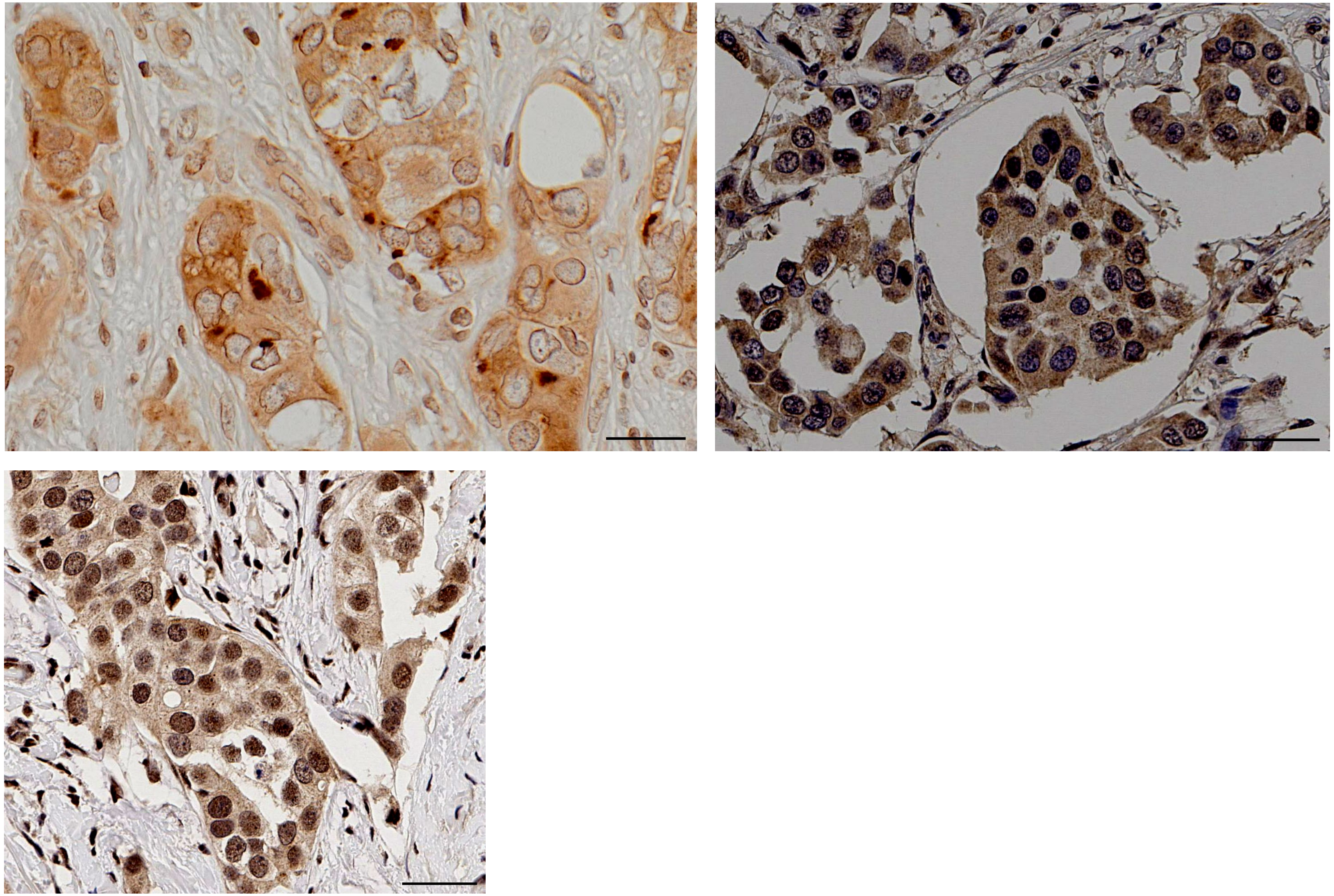

IDC

All Scale Bars are 50 µm
